# Understanding China’s growing involvement in global health and managing processes of change

**DOI:** 10.1186/s12992-020-00569-0

**Published:** 2020-05-01

**Authors:** Lewis Husain, Gerald Bloom

**Affiliations:** Institute of Development Studies, Library Road, Brighton, BN1 9RE UK

**Keywords:** China, Global health, Global governance, Complexity, Change management, Experimentation, Mutual learning

## Abstract

**Background:**

Recent years have seen a rapid change in China’s global engagement and a recognition that solving global challenges will need to take the changing role of China into account. The paper discusses China’s growing involvement in global health. Health is an area where there is broad agreement over global priorities and, potentially, a fertile space to build new forms of collaboration that point the way towards the adaptation of global governance to a rapidly changing context.

**Results:**

Drawing on previous analyses of China’s management of change in its domestic health reforms and interviews with a range of stakeholders in China, the UK and Switzerland, the paper argues that China’s engagement in global health is developing and diversifying rapidly in response to the central government’s desire to see a greater role for China in global health. This diversification is part of a pattern of change management familiar from China’s domestic reform experience. Explorations underway by a range of Chinese agencies form part of a process of rapid experimentation and experiential learning that are informing China’s search for (a) new global role(s).

**Conclusions:**

China is undergoing rapid institutional innovation and developing capacity for greater global engagement, including in health; however, substantial, recent leadership commitments make clear Chinese agencies’ need for continued exploration, innovation and rapid learning. How China engages globally is of significance to the world, not just China. The challenge for China, other global actors and multilateral organisations is to incorporate new approaches into existing global governance arrangements, including for the management of global health. This will require a willingness on all sides to learn from each other and invest the effort needed to build governance arrangements appropriate for the coming decades. This is not only important as a means of protecting global public health, but also as a demonstration of how governance arrangements can be adapted to the needs of a pluralistic global order in a context of rapid change.

## Background

There is a growing recognition that efforts to address global challenges will need to take the changing role of China into account. Many of these challenges involve the negotiation of complex conflicts of interest and it is difficult, in a context of intensifying global economic competition, to imagine how countries will begin to resolve them. The case of health may be different because there is already a broad global consensus on aims, expressed in the sustainable development goal of universal health coverage and agreement on the importance of effective surveillance and response to disease outbreaks. The COVID-19 outbreak has made the need for global cooperation abundantly clear.

The aim of this paper is to identify challenges that China and other countries will need to address in building this kind of cooperation. It brings together two sources of data. The first is a series of analyses by this paper’s authors of China’s approach to the management of change in its own health system [1–4]. The second is a review of documents and a number of interviews undertaken as part of an evaluation of the UK Department for International Development-funded China–UK Global Health Support Programme (GHSP).^1^ These interviews provided a unique opportunity to gain an understanding of the perspectives of the leaders of China’s rapidly expanding global health sector and of global health actors with whom they are engaging. Section 2 provides more information on the data collected. The paper argues that China is taking a similar approach to managing its changing engagement in global health as in its own health sector and that efforts to build cooperation need to take this into account.

The remainder of the paper is structured as follows. The next section outlines the context of China’s experience of rapid development and the implementation of major health system reforms. It focuses particularly on China’s approach to the management of health system adaptation to rapid change. The following section outlines the increasing engagement by the Chinese Government in international development and global health. It describes how the government and other agencies are building capacity to translate ambitious policy statements into action, at scale. The paper concludes with a discussion of the challenges for China and other global health actors in establishing mechanisms for effective collaboration in a rapidly changing global context.

### China’s domestic development – failing to fail

China’s development, since it began its transition from a command economy in the late 1970s, has been rapid and sustained. Despite persistent predictions of system collapse, the country has “failed to fail” [5]. It has experienced huge economic and industrial restructuring, changing from a poor and predominantly agrarian economy to a more diversified, higher-wage and increasingly consumption-based economy. Sustained growth of GDP, at around 10% per year between the early 1980s and early 2010s, has supported increases in welfare, including long-run increases in incomes and reductions in poverty of “unprecedented … scope and scale”, contributing around 70% of global reduction of the number of people in extreme poverty between 1990 and 2013 [6]. China’s achievement of the Millennium Development Goals, on poverty reduction and improvements in health was a major contributing factor to their achievement globally [7]. This success has led to a belief by Chinese policy-makers and researchers that their country’s experience has major lessons for others [8, 9]. It has also fostered an optimistic willingness to engage in ambitious and rapid processes of global development and change. This optimism underlies the major international initiatives we describe in section 2.

China has experienced improvements in health, including in life expectancy, infant and maternal mortality, vaccine coverage, and in the provision of health services, including numbers of health facilities, beds, and trained health workers [6]. One example of the improvements in health is the fall in maternal mortality from 58 to 29 per 100,000 births, between 2000 and 2017 [10]. In recent years, China has also developed (or re-developed) social protection schemes, including health insurance schemes, which now cover around 95% of the population and are being merged and extended to support greater coverage and protection [11].

Many challenges persist and new ones continually emerge. Problems associated with residual poverty, high levels of inequality, rapid increases in the cost of health services, substantial environmental degradation, emergence of infectious diseases, and major social strains associated with the pace of change remain. New challenges include adapting to climate change, the emergence of antimicrobial resistance, an ageing population and a rapid increase in the burden of non-communicable diseases. Limited accountability and sometimes insufficient regulation (or enforcement) remain problematic, including in the health system [6]. The government needs to address these issues at the same time as it attempts to rebalance the economy towards slower, greener and more inclusive growth.

### Chinese government approaches to change management

There is an increasing recognition of the significance of China’s growth and transformation since the 1970s and analysts have put forward a number of explanations of what has made China’s ‘model’ of development distinct [12, 13]. This has led to an interest in China’s management of change. Since the commencement of the transition from a command economy, government policy has been dominated by the over-riding imperatives of ensuring rapid economic development and avoiding major policy errors that could provoke social disorder. An important strategy for achieving these imperatives has been the encouragement of policy experimentation and innovation. A number of analyses have pointed to the importance of experimentation in testing novel policy solutions before large-scale adoption to learn about effectiveness and reduce the risk of unintended and deleterious outcomes [14, 15]. These approaches have helped China ‘discover’ and evolve appropriate institutions and policy approaches to underpin development [6, 16]. One example is the way that the government encouraged localities to test alternative approaches for establishing some form of rural health insurance before implementing a nation-wide reform in 2009 that included rural health insurance [1, 2]. Analysts point out functional similarities between Chinese approaches to experimentation as a component of the management of rapid change and approaches now being recommended by the global development community [4, 6].

Chinese policy experimentation and innovation encompasses a range of phenomena including ‘directed experimentation’ that is overseen or managed centrally, and ‘organic innovation’. In the latter, implementing units (often local governments) respond to signals in policy and leadership speeches, and government incentive structures, and align their reforms to the general policy ‘direction’, giving rise to a range of policy practices. These innovation and experimentation processes are often quite pluralistic, involving not just government but also research agencies, universities and international organisations [17, 18]. This produces a range of practices and outcomes, including non-optimal ones; nonetheless it helps support system adaptation.

The significance of experimentation and innovation in the Chinese policy repertoire poses questions about how it functions (and can be made to function better) as a mechanism for learning. There is increasing evidence that experimentation can help underpin adaptation, through the promotion of ‘appropriate’ institutions with a high degree of contextual fit [19]. This kind of adaptation is necessary to support institutional adaptation in a ‘non-ergodic’ world, i.e. a constantly changing context in which problems are continually ‘novel’, requiring fresh solutions for which we have no exact template [20, 21].

Set against this, there is less clear understanding of *how* to learn from dispersed innovation and experimentation under such conditions. As Dani Rodrik has phrased the question, “We shall experiment, but how shall we learn?” [22]. Historically, the Chinese polity has made use of a number of mechanisms to support intra-systemic learning, including government meeting cycles to continually review and adjust policies [23], field visits and inspections, use of policy briefings and media to disseminate practices judged to be useful and to encourage mutual learning, rotation and secondment of government officials and so forth [3]. Research institutes and universities have played a significant role in this process, helping to uncover, assess and propagate potentially significant or useful practices from among the range of practices produced at times of rapid reform. As China has established increasingly complex institutional arrangements it has begun to formalize evaluation and learning processes, leading to a growing interest in contemporary monitoring and evaluation methods [24]. In the following section we argue that China is replicating this strategy for managing adaptation to rapid change as it substantially increases its capacity to become an effective partner in global development and global health.

### China’s increasing engagement in global health

This section draws on reviews of English and Mandarin-language policy documents and policy analyses and around 300 interviews between 2016 and 2018 with around 150 informants. The interviewees were based in a range of organisations, including several Chinese government agencies working on international development and global health, Chinese universities and research institutions and counterparts in government and research organisations in the U.K., and in international organisations located in China and Geneva. In addition, we interviewed people involved in Chinese pilot projects supported by the GHSP in Tanzania, Ethiopia and Myanmar and development partners in these countries. We also attended a number of GHSP-supported project design meetings, workshops, and formal meetings over the course of the programme. The interviews focused on the respondents’ understandings of the policy priorities, activities undertaken and the lessons that have been learned concerning China’s involvement in both international development and global health and international collaborations. The interviews were not recorded to encourage frankness and the notes were used in compiling the reports. A review of ethical issues was included in the inception phase of the evaluation.

### International development and global health

Historically, China’s engagement in global health has been relatively distinctive. Its health aid mainly consisted of sending medical teams overseas, building health facilities, donating drugs and equipment, and providing some support for malaria control activities. These activities have mostly taken place on a bilateral basis. Since the early to mid-2000s, the country has increased its engagement with multilateral and global bodies, and has increased its support for training, including training of foreign health professionals in China [25]. Preventing the spread of infectious diseases into China, including through cross-border, regional and global mechanisms, has long been a concern of the Chinese government [26, 27].

Since the early 2010s, the Chinese leadership has increasingly promoted a new vision of China’s engagement in the global system, and in international development. Support for global health is part of this new agenda. We list several issues and discuss them in the text that follows.

- The Chinese leadership envisages a substantial change in China’s international engagement, building on lessons from its recent development experience. The intellectual framework for this is being articulated in leadership speeches and in authoritative publications.
- A greater Chinese role in international development, broadly conceived, is being supported by the creation of a range of new institutions and the commitment of substantial funds.
- China is increasing its overseas health commitments and including discussions of health in a number of international, regional and global fora.
- China’s de facto international and global health engagement is diversifying rapidly, though the range of initiatives now emerging is not highly coherent, reflecting the speed of change and the need for Chinese institutions to find new roles in global health.

#### Leadership commitment to a new role

Major speeches by President Xi Jinping at key international fora signal the Chinese leadership’s determination to play a more central role in global affairs, supported by rhetorical innovations such as forging a ‘global community of common destiny’ [28]. The Belt and Road Initiative presents a leadership vision (and a rhetorical umbrella) for increased cooperation with a very large number of countries, in multiple sectors. It also reflects a belief on the part of the Chinese leadership that China’s experience of rapid economic transformation has important implications for global development. Development assistance to other developing countries and south-south cooperation have been written into major national plans and strategies, including China’s thirteenth five-year plan [29]. Domestic strategies and speeches make clear that the leadership intends China to play a more central (and active) role in global governance, one more fitting to the country’s self-image as a major power [30]. This coincides with analysis within the foreign policy community, and theoretical innovation regarding China’s global role [31]. High-profile leadership visits to low-income countries, including many African countries, have increased.

#### New forms of support for a greater global role

Alongside this increasing rhetorical commitment, China has supported the establishment of new institutions and financing instruments to play a greater role in international development, including the Asian Infrastructure Investment Bank, the New Development Bank (the ‘BRICS Bank’), the Silk Road Fund, the UN Peace and Development Fund [32], and the South-South Assistance Cooperation Fund [33]. In recent years, China has also made commitments to provide assistance through existing channels such as the Forum on China-Africa Cooperation (FOCAC). Chinese nationals are increasingly present in the UN system [34]. In early 2018, the China International Development Cooperation Agency (CIDCA) was established, as part of a process of developing a more consolidated and institutionalised system to support a greater Chinese role in overseas assistance. Until then, aid was organized by sectoral ministries with the Ministry of Commerce (MOFCOM) having overall responsibility. For example, the National Health Commission (NHC) has organized the deployment of medical teams to other countries, while MOFCOM has been responsible for financing the construction of health facilities, the supply of drugs and so forth. At the same time, the government has encouraged research institutes that have been deeply engaged in supporting China’s internal process of rapid change, to establish think tanks to facilitate mutual learning between China and other countries.

#### Increasing commitments to health assistance and cooperation

China’s commitment to global health has increased over the past ten or more years. Frameworks for collaboration with African countries, such as FOCAC, have consistently included health cooperation in their remit. A forum for China-Africa Health Development has been included under FOCAC, and there have been commitments to provide health assistance to African countries [35]. Similar evolutions can be seen in a number of south-south fora, such as the BRICS (Brazil, Russia, India, China, South Africa), Asia-Pacific Economic Cooperation, G20 and the Association of Southeast Asian Nations. In 2017, a Joint Communiqué of The Belt and Road Health Cooperation and Health Silk Road was issued, setting out areas in which China seeks to work with other countries along the Belt and Road [36]. The NHC, formerly the National Health and Family Planning Commission, China’s health ministry, has issued two strategies for Belt and Road health cooperation, to systematise and guide country-wide health cooperation.^2^ The Government has signed memoranda of understanding (MOUs) with a number of multilateral organisations as part of the process of increasing global cooperation, most notably for global health with the WHO [38]. In 2016, the NHC developed an internal global health strategy to guide its work [39]. Taken together, these documents, strategies and policies show commitment to an increased role in global health and start to show the thematic priorities and forms of cooperation and engagement that the government envisages.

#### Diversifying health engagement

Various analyses show an increase in China’s funding for overseas health assistance. Reflecting different methods and data, these analyses give different figures for the scale of the increase, though there is little doubt that China’s health assistance is increasing rapidly – one recent Chinese analysis concluded that China’s health assistance has been growing at an average annual rate of 121% since 2003, but from a very low base [40].

While much spending continues to be on constructing health facilities, China’s overseas health engagement is diversifying and becoming more complex. Alongside high-profile commitments such as financing the construction of the Africa Centre for Disease Control and Prevention (CDC) and their five regional sub-centres (and staff secondments to the CDC) [41], support for several direct interventions overseas,^3^ and programmes such as the Brightness Project [45], many less prominent initiatives are underway. Examples include the continued support by the Chinese CDC for public health strengthening in Sierra Leone, a range of new bilateral cooperation agreements,^4^ diversification of the functions of Chinese medical teams, supporting Chinese emergency medical teams to get WHO-prequalification, and support for joint external evaluations in several Asian countries.^5^

The National Health and Family Planning Commission Belt and Road Health Cooperation Plan issued in 2015 [37] provides a snapshot of the breadth of activity underway, including commercial activity, health assistance, infectious disease control and health security, primarily framed as ‘cooperation’ rather than ‘assistance’. It also shows a number of new drivers (including soft power promotion and research and development collaboration) and innovations in approach (including linking of global and domestic developmental challenges, application of new technologies, and mobilization of ethnic Chinese identifies in linking with, for example, the Arab World). Reflecting substantial decentralisation in China’s management of health and social welfare [48], as well as the distributed nature of much of China’s external engagement [49], the activities profiled in the plan are a mixture of mandated, top-down initiatives and comparatively bottom-up initiatives developed by provinces or sub-national agencies in response to the overall direction of national strategy and policy. Equally, and in line with the transformation that China’s overseas engagement is undergoing, multiple health-related agencies are developing their own strategies for overseas engagement related to their own areas of expertise.^6^

This rapid increase in national commitment, and the proliferation of initiatives that is starting to become visible, have the following implications for global health:

- First, China’s engagement in global health is occurring against the background of a major shift in the country’s international engagement, and debate (inside and outside China) about China’s future global role.
- Second, while China’s overall global health engagement has historically been relatively distinct, its new role has yet to be defined clearly. China has no template to guide this, but many agencies are actively exploring new roles. As can be seen from the linking of multiple kinds of activity such as assistance, research and development and commercial activity, China’s role(s) is (are) unlikely to conform neatly to practices of existing donors and major global health agencies.
- Third, most Chinese agencies (including government and research agencies) have little experience of carrying out health interventions or collaborations overseas. They face a steep learning curve as they experiment with new approaches.
- Fourth, as China becomes increasingly involved in global health, this will have important implications for current arrangements for the governance of this sector.

### Building capacity for increased engagement in global health

This sub-section focuses on the activities supported by the GHSP during 2014–2018. Increasing leadership attention and commitment has led to the rapid proliferation of global/international health-related initiatives across the Chinese system. Many agencies with health remits are now looking for new roles that reflect their strengths and experience, while extending their work outside China’s borders. This process of proliferation has been common during China’s domestic reforms but is now starting to play out internationally. It is, necessarily, both a process of exploring new practical approaches (to health assistance, south-south collaboration, and so forth) as well as a process through which individuals and agencies build capacity and understanding of global health, and China’s – and their – place in it. The GHSP provided space for this kind of exploration, reimagining of China’s global health engagement, and development of individual and institutional capacity.

The strategy adopted by the GHSP closely mirrored approaches used by the Chinese government to build capacity to oversee and support domestic health system adaptation to rapid change and health system reforms. The project was overseen by a Strategic Oversight Committee with representatives from DFID, the NHC and MOFCOM, with practical management by a project management agency affiliated with the NHC. The strategy implemented between 2013 and 2018 involved:

- Building the capacity of government to oversee a major increase in global health engagement by supporting targeted analysis and support to strategy/policy development;
- Establishing think tanks and networks with expertise to provide advice to government and other stakeholders and support learning;
- Testing implementation of new programmes and building capacity to implement them at scale and
- building engagement with partners such as the UK, WHO, and governments of countries in which programmes will be implemented.

Some of the key findings concerning each of the above elements of the strategy are summarized below. Many have been reported in the programme annual reviews.^7^

#### Building government capacity

The programme employed several strategies for building the capacity of government to support increased Chinese engagement in global health. A number of government departments have been responsible for different aspects of global health and international development assistance. These include the National Development and Reform Commission, which oversees large investments, the Ministry of Finance, the Ministry of Foreign Affairs, which manages relationships with other countries, the Ministry of Commerce, which manages development assistance, and the National Health Commission. The roles and responsibilities of many government agencies are in flux as the government system undergoes rapid change and as China’s international engagement changes gear. As during other periods of significant system reform in China, there will be a period of accommodation *en route* to a new stable configuration. The GHSP provided support to a leading Chinese university to coordinate analyses, in conjunction with the NHC, that would contribute to development of a global health strategy for that agency. The GHSP also supported a number of leading Chinese research institutes and universities to carry out analyses of a range of issues relating to China’s global health engagement, and to translate much of this into policy briefings that were shared within the NHC and, to an extent, more widely.^8^

The programme also supported courses on global health and global health governance for government officials and leaders in relevant research agencies implementing health programmes aimed at building capacity and creating links and shared perspectives between agencies. The GHSP also provided opportunities for exposure to a range of global and overseas health activities, including through a small number of secondments, visits to Chinese-supported overseas health pilot programmes, and dialogue with multilateral and UK agencies (principally DFID, through an annual dialogue). The consensus of the informants was that these activities were useful in starting to build an understanding of many aspects of global health, but that more will be needed to create capacity for substantial interventions in global health.

#### Creating capacity to provide expert advice

Universities and research institutes have played an important role in China’s domestic health system reforms, by identifying problems to be addressed, developing innovative solutions in partnership with local governments, evaluating innovations and providing expert advice to government departments. Along with China’s pivot towards greater engagement in global health, universities and research institutes are establishing centres for global health that will provide similar support for substantially increased engagement in global health. With funding from the GHSP, a number of universities and research institutes reviewed China’s experience with several large-scale programmes, to control schistosomiasis and malaria, reduce maternal and child mortality, and the country’s recent experience with large-scale health sector reform and produced publications for an international readership. Researchers from those institutions have been invited to provide expert advice to government agencies as demand increases for understanding of how China can create a new global health role. A number of networks focusing on global health are now emerging The Chinese Global Health Network, supported by the GHSP, provides a forum linking Chinese researchers, government agencies and third sector and private entities working on global health. To date there has not been a clear decision from Chinese research funding bodies regarding how China will finance research and technical support for global health. Much research continues to rely on ad hoc funding and funding from non-Chinese sources. Some new funding sources, such as the South-South Cooperation Assistance Fund (SSCAF) could create opportunities for projects that will involve Chinese researchers in work on global health issues. The Chinese government has increasingly high expectations for China’s overseas development engagement, and this will require broad capacity in research and policy support.

#### Learning from small-scale interventions

Several small-scale pilot interventions were included in the GHSP to test how elements of China’s experience could be adapted to support the design and implementation of interventions in other countries. These interventions were led by Chinese health implementation agencies, including an affiliated agency of the Centres for Disease Control and university research centres. Chinese experts worked with partners in other countries to design and implement interventions that drew on Chinese experience. Visits by one of the authors to some pilot sites found that they provided an opportunity for several high-capacity Chinese institutions to develop interventions overseas, experiment with translating Chinese experience in new contexts and explore new kinds of assistance projects that China could support as it diversifies its health assistance. The pilots revealed many of the challenges that implementing agencies will need to address if China decides to establish its own capacity to undertake major health system strengthening and reform projects at scale. These included common problems that arise when working in another country. The signing of MOUs with multilateral organisations such as the WHO, and the establishment of new funding sources such as the SSCAF, show that the government is still exploring the appropriate balance between strengthening capacity within China to design, implement and evaluate major multi-country health system development programmes, collaborating with other bilateral agencies and working through multi-lateral organisations and global initiatives.

#### Strengthen collaboration with global partners

Senior officials from Chinese and UK agencies met annually through a bilateral Global Health Dialogue. This provided an opportunity to identify areas of common interest for future collaboration. A few modest spin-off collaborative initiatives were in evidence by the end of the programme. The Chinese Government has also signed MOUs with the WHO and with several bilateral partners and is providing support to the Africa CDC. All these initiatives are still at an early stage. These experiences highlighted some constraints to translating agreements into substantial programmes of collaboration with bilateral and multi-lateral development organizations. One is the need to involve all relevant government departments such as International Development, Health, Commerce, Finance and Foreign Affairs. China and the UK are strengthening coordination at national level, however mechanisms to make this effective in bilateral cooperation are not firmly in place. This could be addressed by the identification of government focal points to manage bilateral cooperation on global health. A second constraint is the need to facilitate joint working between government administrative systems in China, bilateral partners and multi-lateral organizations. This will involve significant efforts by counterpart agencies involved in the funding and management of global health activities. A third constraint is the thinness of existing cooperation between China and international partners. This needs to involve health research organisations, agencies that manage international development programmes, private health sector companies and so forth. There are already examples of effective collaboration, however this needs to be deepened and mechanisms are needed for governments to mobilize this expertise in coordinated efforts to address public health challenges. This could involve the creation of a forum for global health collaboration. One aim would be to build mutual understanding between technical experts as a basis for building more effective collaboration.

## Conclusions: experimentation and innovation for global health

In the late 1970s, as China started to launch market reforms, it did so in a pragmatic way, which allowed experimentation and learning about what might work in its distinct institutional, political and economic environment. In doing so, the country adopted a number of unorthodox approaches and started a process of gradual and experimental transition to a new (though not finally or fully defined) state. Experimentation has played an important role in subsequent reforms to the economy, state, political and administrative structure, and society. This has enabled China to achieve rates of development that would have been considered by almost all experts to be impossible. A similar process at the global level is now starting as the leadership encourages a rapid increase in China’s role in international development, including health. As with so many rounds of previous reforms, high-level *aims* (targets and aspirations) are set out in national strategies and leadership speeches. These encourage implementing agencies to carry out widespread experimentation and innovation to create the *means* needed to achieve them.

Unpacking the concept of *means* shows the necessity of experimentation, innovation and learning on multiple fronts. As outlined above, this is already taking place in areas including exploration of China’s historical approaches to health assistance, reflection on the usefulness of these approaches in the early twenty-first century, and their significance given the new context of China’s rapidly increasing engagement and debates regarding what China’s new global role should be.

These experiences have shown the need for widespread development of capacity to perform the kinds of tasks (for example in research, analysis, support to decision- and policy-making) required by China’s increasing global health engagement. They have also shown the need for new kinds of linkage between agencies, including various functional networks (e.g. networks linking universities developing courses and research on global health), links between government and non-government agencies (e.g. between the NHC and research agencies), between different parts of government (e.g. the NHC and new agencies such as CIDCA) and between Chinese agencies and international partners (e.g. WHO, DFID).

Many Chinese health agencies are increasing their capacity to provide the new kinds of analysis needed by decision makers. Initiatives such as the 2015–2017 BRI health cooperation plan show how highly pluralistic, emergent initiatives across the country are being screened and assessed for their usefulness as potential models for a more diversified Chinese role in global health. CIDCA and the SSCAF are institutional innovations intended to support new initiatives by Chinese and external agencies (notably UN agencies) and explore new cooperation modalities outside the constraints of the current Chinese institutional system [50]. However, this is only a beginning. These agencies still employ a small number of people with the expertise and experience they need. This is likely to change over the next few years.

The breadth of recent leadership commitments makes clear the need for continued exploration, innovation and learning if Chinese agencies are to fulfil the ambitions of the leadership for greater engagement. As we have argued, China has historically employed approaches to rapid experimentation and learning in managing domestic reforms. Approximately five years on from the beginning of implementation of the GHSP, there is now considerable ferment in China’s global health engagement and a proliferation of new initiatives.

How China engages globally is of significance to the world, not just China. Chinese engagement – for example in Africa – most recently badged as Belt and Road Initiative, has stimulated a great deal of analysis. There is criticism, as well as analyses that point to the positive potential of China’s changing global engagement [51]. Countries typically have a variety of motivations for engaging in development cooperation and global health including geopolitical, commercial interests and domestic political concerns as well as a recognition of the importance of certain global public goods. The actions of China will increasingly have an impact on global health and that country will, in turn, be increasingly exposed to risks associated with its dense networks of global communication. These are important issues that merit additional research and analysis. This paper does not attempt to assign weights to the different motivations of global health actors. Its focus is on areas of widespread agreement on global health objectives and on the serious negative consequences of a failure to cooperate. It argues that a significant effort will be needed to build effective collaboration and avoid these deleterious outcomes.

China’s engagement in global health comes at a time of very rapid change as rapid global development leads to new public health challenges, while a number of developed countries either retrench or refashion their assistance portfolios. There is little doubt that there is a need for increased resources and new solutions to global health problems. The Chinese leadership is intent on greater cooperation in this area, and programmes such as the GHSP show the significance attached by other countries, UN agencies and the like to work with China as an emerging actor in development. The medium- to long-term outcomes of such collaborations and China’s increasing engagement in development and health will be strongly influenced by the willingness and capacity of Chinese institutions to learn from these new experiences and adapt their policies and practices, as well as by the willingness and capacity of ‘incumbent’ agencies to adapt.

Recent years have seen an increase in attention to the rise of new development actors and the distinct developmental experience or approaches they bring, as well as the increasing significance of south-south learning and policy transfer. Much of the focus has been on policy transfer and learning at the level of particular practices or policy models. This paper has discussed a higher-level phenomenon – approaches that are not specific to one particular technical area, but concern system-level institutions or rules that guide the ways that change is managed.

The 2015 World Development Report pointed out many heuristics and biases of the global development community, arguing that these colour the ways that development professionals see the world, its problems, and potential solutions [52]. With the increasing engagement of countries such as China in global development, the ‘development community’ will surely become more diverse, bringing a wider range of world views to bear when trying to solve problems. As development and global health debates diversify, there is increasingly a need for new forms of understanding – for Chinese development and global health professionals to understand the global system and how to engage in it, and for professionals from established donor countries, multilateral agencies and low and middle-income countries to better understand the approaches, thinking and development experiences that their new peers bring.

Practically speaking, there is a need for experimentation and learning in the context of the rapid social, economic and environmental change that is currently underway [53]. The major investments funded as part of the Belt and Road Initiative are likely to strengthen and deepen regional and global connectivity, resulting in new public health challenges – and new possibilities for addressing them. The GHSP provided modest levels of support for an initial stage of learning that has principally helped inform change in China. As China’s global footprint increases, there is a need for initiatives that create the learning needed by multiple partners and agencies regarding how to work together to deal with global challenges. Several programmes, including the GHSP, have started to provide evidence on how experimental initiatives can foster this kind of learning. There is now a need for initiatives and substantial investment in new approaches to learning about the management of change. Potential bilateral and multi-lateral partners will need to make a substantial effort to build their capacity to collaborate effectively with China, as China will need to build capacity to collaborate with others. It may take a lot of time and effort to agree on global decision-making processes and, meanwhile, effective responses to disease challenges could be delayed. However, the alternative would be a long-term fragmentation of public health efforts.

The emergence of a new global power inevitably disrupts established ways of doing global governance. This is taking place in a period of rapid change that is presenting major challenges that require global cooperation. The challenge for China, other global actors and multilateral organisations is to find ways to incorporate new approaches to global collaboration, while maintaining the stability of existing governance arrangements for global health. This will require a willingness on all sides to learn from each other and invest the effort needed to build governance arrangements appropriate for the coming decades. This is not only important as a means of protecting global public health, but also as a demonstration of how governance arrangements can be adapted to the needs of a pluralistic global order in a context of rapid change. This is the challenge the new emerging global community faces as it attempts to build a cooperative approach for controlling the COVID-19 pandemic.

## Data Availability

Data sharing is not applicable to this article as no datasets were generated or analysed during the current study.

## Abbreviations

Africa CDC: Africa Centre for Disease Control and Prevention (CDC
CIDCA: China International Development Cooperation Agency
DFID: United Kingdom Department for International Development
FOCAC: Forum on China-Africa Cooperation (FOCAC).
GHSP: China–UK Global Health Support Programme
MOFCOM: Ministry of Commerce of the People’s Republic of China
MoU: Memorandum of understanding
NHC: National Health Commission of the People’s Republic of China
SSCAF: South-South Cooperation Assistance Fund
UN: United Nations
WHO: World Health Organisation

## Footnotes

1 Lewis Husain was evaluation team lead, and Gerald Bloom was involved over the course of the evaluation, including in shaping design, data collection and analysis and drafting reports. The evaluation was carried out by Itad (link) through the ePact Consortium, and was funded by the UK Department for International Development.

2 The first was available through the NHFPC website [37]. The second is not publicly available.

3 Notable examples are pilot projects in Tanzania, Myanmar and Ethiopia supported through the GHSP (see GHSP project completion review [42]), a Sino-Australian collaboration on building capacity for malaria control in Papua New Guinea [43], and a malaria eradication initiative in Comoros [44].

4 Examples include agreements such as the Sino-Czech 2017–2020 health cooperation plan [46], announcement of health as a priority in collaboration with the Lao PDR [47], and support to development of public health institutes in Myanmar and Cambodia.

5 A list of JEE mission reports for the WPRO region is available here. A list of WHO-classified EMTs is available here.

6 See, for example, the 2016–2020 plan for development of Chinese medicine and drugs in the BRI (*Zhongyiyao Yi Dai Yi Lu fazhan guihua, 2016–2020 nian)* (available here), and the 13th five year plan for development of family planning in China, which emphasises the need for increased international collaboration, including south-south collaboration, and collaboration in countries participating in the BRI (*Shisanwu quanguo jihua shengyu shiye fazhan guihua)*, available here.

7 Annual reviews were carried out in every year of the programme’s operation, noting progress in implementation against defined outputs and outcomes, the extent to which implementation was in line with the programme theory of change, and providing recommendations for tailoring implementation. A project completion review was carried out in 2019. Annual reviews and the project completion review can be found on the DFID website (link).

8 The GHSP supported the production of a large amount of research (more than 80 peer reviewed articles and book chapters in English and Chinese; DFID 2019), as well as more than 40 policy briefs (link).
